# Calibration Belt for Quality-of-Care Assessment Based on Dichotomous Outcomes

**DOI:** 10.1371/journal.pone.0016110

**Published:** 2011-02-23

**Authors:** Stefano Finazzi, Daniele Poole, Davide Luciani, Paola E. Cogo, Guido Bertolini

**Affiliations:** 1 Astrophysics Sector, Scuola Internazionale Superiore di Studi Avanzati and Instituto Nazionale di Fisica Nucleare Sezione di Trieste, Trieste, Italy; 2 Intensive Care Unit, Department of Anesthesia and Intensive Care, San Martino Hospital, Belluno, Italy; 3 GiViTI Steering Committee, Italy; 4 Unit of Clinical Knowledge Engineering, Laboratory of Clinical Epidemiology, ‘Mario Negri’ Institute for Pharmacological Research, Milano, Italy; 5 Pediatric Intensive Care Unit, Department of Pediatrics, Padova University, Padova, Italy; 6 Laboratory of Clinical Epidemiology, GiViTI Coordinating Center, ‘Mario Negri’ Institute for Pharmacological Research, Ranica, Italy; University of Swansea, United Kingdom

## Abstract

Prognostic models applied in medicine must be validated on independent samples, before their use can be recommended. The assessment of calibration, *i.e.*, the model's ability to provide reliable predictions, is crucial in external validation studies. Besides having several shortcomings, statistical techniques such as the computation of the standardized mortality ratio (SMR) and its confidence intervals, the Hosmer–Lemeshow statistics, and the Cox calibration test, are all non-informative with respect to calibration across risk classes. Accordingly, calibration plots reporting expected versus observed outcomes across risk subsets have been used for many years. Erroneously, the points in the plot (frequently representing deciles of risk) have been connected with lines, generating false calibration curves. Here we propose a methodology to create a confidence band for the calibration curve based on a function that relates expected to observed probabilities across classes of risk. The calibration belt allows the ranges of risk to be spotted where there is a significant deviation from the ideal calibration, and the direction of the deviation to be indicated. This method thus offers a more analytical view in the assessment of quality of care, compared to other approaches.

## Introduction

Fair, reliable evaluation of quality of care has always been a crucial but difficult task. According to the classical approach proposed by Donabedian [1], indicators of the structure, process, or outcome of care can be variably adopted, depending on the resources available, the purpose and the context of the analysis. Whichever indicator is adopted, quality of care is assessed by comparing the value obtained in the evaluated unit with a reference standard. Unfortunately, this approach is hampered by more or less important differences between the case-mix under scrutiny and the case-mix providing the reference standard, thereby precluding direct comparison. To solve this problem, multipurpose scoring systems have been developed in different fields of medicine. Their aim is to provide standards tailored on different case-mixes, enabling the quality of care to be measured in varying contexts. Most of these systems are prognostic models, designed to estimate the probability of an adverse event occurring (*e.g.*, patient death), basing quality of care assessment on an outcome indicator. These models are created on cohorts representative of the populations to which they will be applied [2].

A simple tool to measure clinical performance is the ratio between the observed and score-predicted (*i.e.* standard) probability of the event. For instance, if the observed-to-expected event probability ratio is significantly lower than 1, performance is judged to be higher than standard, and *vice versa*. A more sophisticated approach is to evaluate the calibration of the score, which represents the level of accordance between observed and predicted probability of the outcome. Since most prognostic models are developed through logistic regression, calibration is usually evaluated through the two Hosmer–Lemeshow goodness-of-fit statistics, 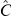 and 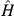 [3]. The main limitations of this approach [4], [5] are overcome by Cox calibration analysis [6], [7], although this method is less popular. All these tests investigate only the degree of deviation between observed and predicted values, without providing any clue as to the region and the direction of this deviation. Nevertheless, the latter information is of paramount importance in interpreting the calibration of a model. As a result, expected-to-observed outcome across risk subgroups is usually reported in calibrations plots, without providing any formal statistical test. Calibration plots comprise as many points as the number of subgroups considered. Since these points are expected to be related by an underlying curve, they are often connected in the so-called ‘calibration curve’. However, one can more correctly estimates this curve by fitting a parametric model to the observed data. In this perspective, the analysis of standard calibrations plot can guide the choice of the appropriate model.

In this paper we use two illustrative examples to show how to fit such a model, in order to plot a true calibration curve and estimate its confidence band.

## Analysis

### Two illustrative examples

Every year GiViTI (Italian Group for the Evaluation of Interventions in Intensive Care Medicine) develops a prognostic model for mortality prediction based on the data collected by general ICUs that join a project for the quality-of-care assessment [8]. In our first example, we applied the GiViTI mortality prediction model to 194 patients admitted in 2008 to a single ICU participating to the GiViTI project.

In the second example, we applied the SAPS II [9] scoring system to predict mortality in a cohort of 2644 critically ill patients recruited by 103 Italian ICUs during 2007, to evaluate the calibration of different scoring systems in predicting hospital mortality.

In the two examples we evaluated the calibration of the models through both traditional tools and the methodology we are proposing. The main difference between the two examples is the sample size: quite small in the former, quite large in the latter example. Any valuable approach designed to provide quality-of-care assessment should be able to return trustworthy and reliable results, irrespective of the level of application (*e.g.*, single physician, single unit, group of units). Unfortunately, due to the decreasing sample size, the closer the assessment is to the final healthcare provider (*i.e.* the single physician), the more the judgment varies. In this sense, it is crucial to understand how different approaches behave according to different sample sizes.

In the first example, the overall observed ICU mortality was 32% (62 out of 194), compared to 33% predicted by the GiViTI model. The corresponding standardized mortality ratio (SMR) was 0.96 (95% confidence interval (CI): 0.79, 1.12), suggesting an on-average behavior of the observed unit. However, the SMR does not provide detailed information on the calibration of the model. For instance, an SMR value of 1 (perfect calibration) may be obtained even in the presence of significant miscalibration across risk classes, which can globally compensate for each other if they are in opposite directions.

**Figure 1:**
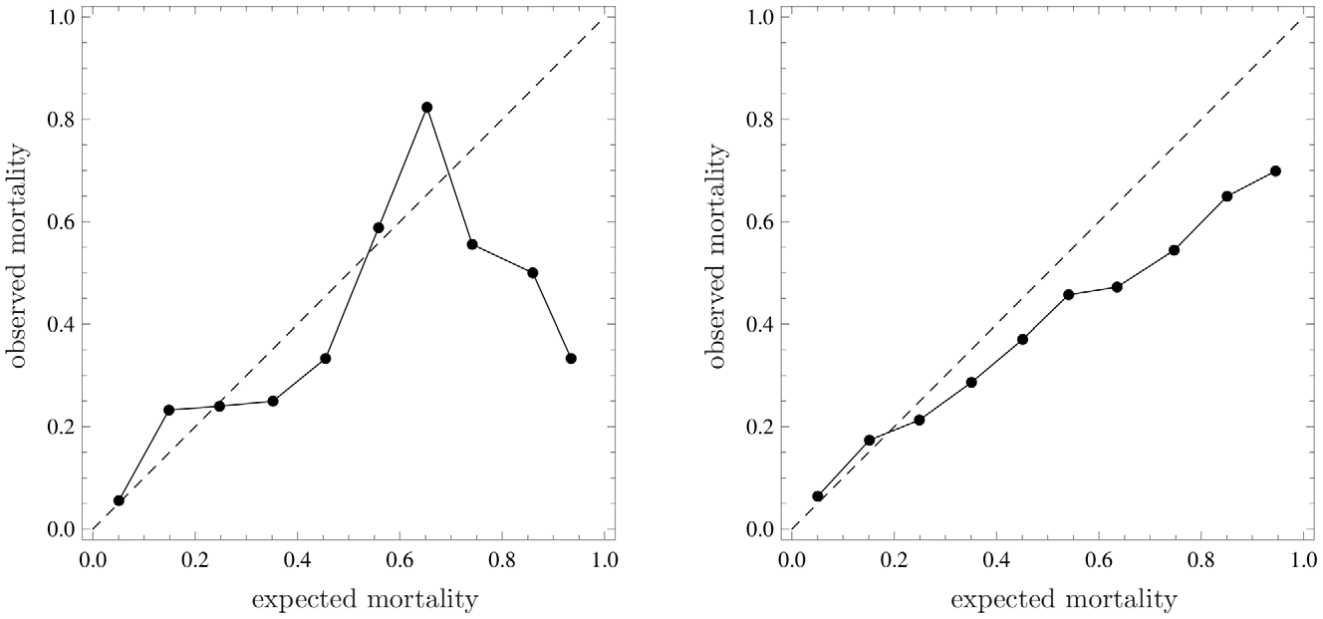
Calibration plots through representation of observed mortality versus expected mortality (bisector, *dashed line*). Left panel: Data of 194 patients staying longer than 24 hours in a single Intensive Care Unit (ICU) taking part in GiViTI (Italian Group for the Evaluation of Interventions in Intensive Care Medicine) in 2008; expected mortality calculated with a prediction model developed by GiViTI in 2008. Right panel: Data of 2644 critically ill patients admitted to 103 ICUs in Italy from January to March 2007; expected mortality calculated with SAPS II.

The Hosmer–Lemeshow goodness-of-fit statistics are an improvement in this respect. In the two proposed tests (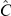 and 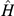), patients are in fact ordered by risk of dying and then grouped in deciles (of equal-size for the 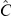 test, of equal-risk for the 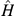 test). The statistics are finally obtained by summing the relative squared distances between expected and observed mortality. In this way, every decile-specific miscalibration leads to an increase in the overall statistic, independently of the sign of the difference between expected and observed mortality. The Hosmer–Lemeshow 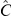-statistic in our sample yielded a 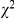-value of 32.4 with 10 degrees of freedom (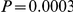), the 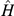-statistic a 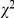-value of 32.7 (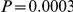). These values contradict the reassuring message given by the SMR and suggest a problem of miscalibration. Unfortunately, the Hosmer–Lemeshow statistics only provide an overall measure of calibration. Hence, any ICU interested in gaining deeper insight into its own performance should explore data with different techniques. More information is usually obtained by plotting the calibration curve (reported in the left panel of Fig. 1), which is the graphical representation of the rough numbers at the basis of the 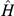-statistic. In the example, the curve shows that the mortality is greater than expected across low risk deciles, lower in medium risk deciles, greater in medium-high risk deciles and, again, lower in high-risk deciles. Unfortunately, this plot does not provide any information about the statistical significance of deviations from the bisector. In particular, the wide oscillations that appear for expected mortality greater than 0.5 are very difficult to interpret from a clinical perspective and may simply be due to the small sample size of these deciles. Finally, it is worth remarking that connecting the calibration points gives the wrong idea that an observed probability corresponding to each expected probability can be read from the curve even between two points. This is clearly not correct, given the procedure used to build the plot.

In the second example, the SMR was significantly different from 1 (0.83, 95% CI: 0.79, 0.88), indicating a lower than expected mortality in our sample. The two Hosmer–Lemeshow goodness-of-fit statistics (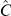-value: 226.7, 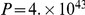; 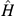-value: 228.5, 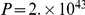) confirm poor overall calibration. Finally, the calibration curve (Fig. 1, right panel) tells us that the lower than expected mortality is proportional to patient severity, as measured by expected mortality. The first two dots are so close to the bisector that they do not modify the general message, despite being above it. Since expected mortality is calculated using an old model, the most natural interpretation is that, as expected, ICUs performed consistently better in 2008 than in 1993, when the SAPS II score was developed.

In summary, the above-mentioned tools for assessing quality of care based on dichotomous outcomes suffer from various drawbacks, which are only partially balanced by their integrated assessment. The SMR and Hosmer–Lemeshow goodness-of-fit statistics only provide information on the overall behavior, which is almost invariably insufficient for good clinical understanding, for which a detailed information on specific values of mortality would be necessary. The calibration curve seems to provide complementary information, but at least two main disadvantages undermine its interpretation: first, it is not really a curve; second, it is not accompanied by any information on the statistical significance of deviations from the bisector. In the following sections, we propose a method to fit the calibration curve and to compute its confidence band. This method is applied to both the examples.

### The calibration curve

We define 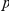 the probability of the dichotomous outcome experienced by a patient admitted to the studied unit and 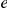 the expected probability of the same outcome, provided by an external model representing the reference standard of care. The quality of care is assessed by determining the relationship between 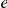 and 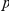 described by a function 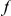. In the ICU example, if a patient has a theoretical probability 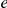 of dying, his actual probability 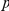 differs from 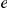 depending on the level of care the admitting unit is able to provide. If he has entered a well-performing unit, 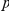 will be lower than 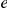 and *vice versa*. Hence, we can write
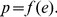
(1)The function 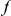, to be determined, represents the level of care provided or, in mathematical terms, the calibration function of the reference model to the given sample.

We start to note that, from a clinical standpoint, 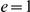 represents an infinitely severe patient with no chance of survival. The opposite happens in the case of 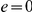, an infinitely healthy patient with no chance of dying. Moreover, in the vast majority of real cases, the expected probability of death is provided by a logistic regression model
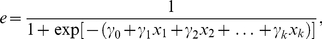
(2)where 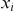 are the patient's physiological and demographic parameters and 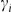 are the logistic parameters. In this case the values 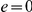 or 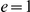 can only be obtained with non-physical infinite values of the variables 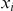, which therefore correspond to infinite (theoretical) values of physiological or demographic parameters.

This feature can be made more explicit by a standard change of variables. Instead of 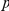 and 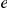, ranging between 0 and 1, we used two new variables 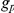 and 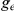, ranging over the whole real axis 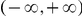, such that 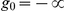 and 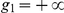. A traditional way of doing so is to log-linearize the probabilities through a logit transformation, where the logit of 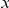 is the natural logarithm of 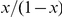. Hence, Eq. (1) is rewritten as
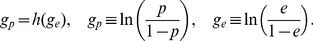
(3)


In a very general way, one can approximate 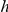 with a polynomial 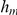 of degree 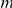:
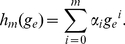
(4)


Once the relation between the logits 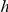 has been determined, the function 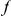, as expressed in Eq. (1), is approximated up to the order 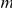 by
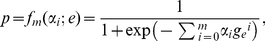
(5)where 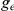 is given in Eq. (3).

When 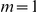, Eq. (5) reduces to the Cox calibration function [6]. In this particular case, the probability 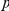 is a logistic function of the logit of the expected probability 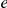. The value of the parameters 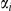 can be estimated through the maximum likelihood method, from a given set of observations 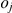, 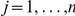, where 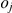 is the patient's final dichotomous outcome (0 or 1). Consequently, the estimators 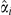 are obtained by maximizing
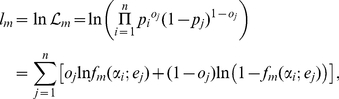
(6)where 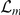 is the likelihood function and 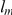 is its natural logarithm.

The optimal value of 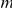 can be determined with a likelihood-ratio test. Defining 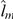 the maximum of the log-likelihood 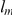, for a given 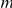, the variable
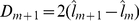
(7)is distributed as a 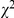 with 1 degree of freedom, under the hypothesis that the system is truly described by a polynomial 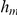 of order 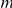. Starting from 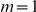, a new parameter 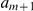 is added to the model only if the improvement in the likelihood provided by this new parameter is significant enough, that is when
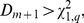
(8)where 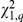 is the inverse of the 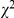 cumulative distribution with 1 degree of freedom. In the present paper we use 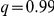. The iterative procedure stops at the first value of 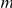 for which the above inequality is not satisfied. That is, the final value of 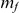 is such that for each 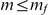, 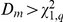 and 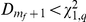.

The choice of a quite large value of 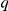 (*i.e.* retaining only very significant coefficients) is supported by clinical reasons. In the quality-of-care setting, the calibration function should indeed avoid multiple changes in the relationship between observed and expected probabilities. Whilst it is untenable to assume that the performance is uniform along the whole spectrum of severity, it is even less likely it changes many times. We can imagine a unit that is better (or worse) at treating sicker patients than healthy ones, but it would be very odd to find a unit that performs well (or poorly) in less severe, poorly (or well) in medium-severe, and well (or poorly) in more severe patients. Large values of 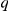 assure to spot only significant phenomena without spurious effects related to the statistical noise of data.

A measure of the quality of care can thus be derived from the coefficients 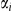. If 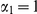 and 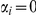 for 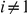, the considered unit performs exactly as the general model (*i.e.*, the calibration curve matches the bisector). Overall calibration can be assessed through a Likelihood-ratio test or a Wald test, applied to the coefficients 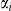, with the null hypothesis 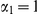, 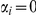 for 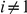, which corresponds to perfect calibration. In the particular case in which 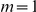, 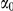 and 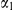 can be respectively identified with the Cox parameters 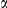 and 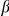 [6]. Cox referred to them respectively as the bias and the spread because 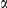 represents the average behavior with respect to the perfect calibration, while 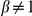 signals the presence of different behaviors across risk classes.

In the first example (single ICU), the iterative procedure described above stops at 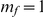, that is the linear approximation of the calibration function. The Likelihood-ratio test gives a 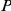-value of 0.048 and the Wald test gives 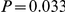. Both tests warn that the model is not calibrating well in the sample. Notably, this approach discloses a miscalibration which the SMR fails to detect (see section *Two illustrative examples*), confirming the result of the 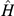 and 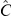 tests. In the second example (a group of ICUs), the iterative procedure described above stopped at 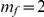. The Likelihood-ratio test gives a 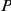-value of 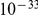 and the Wald test a 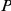-value of 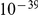, indicating a miscalibration of the model.

**Figure 2:**
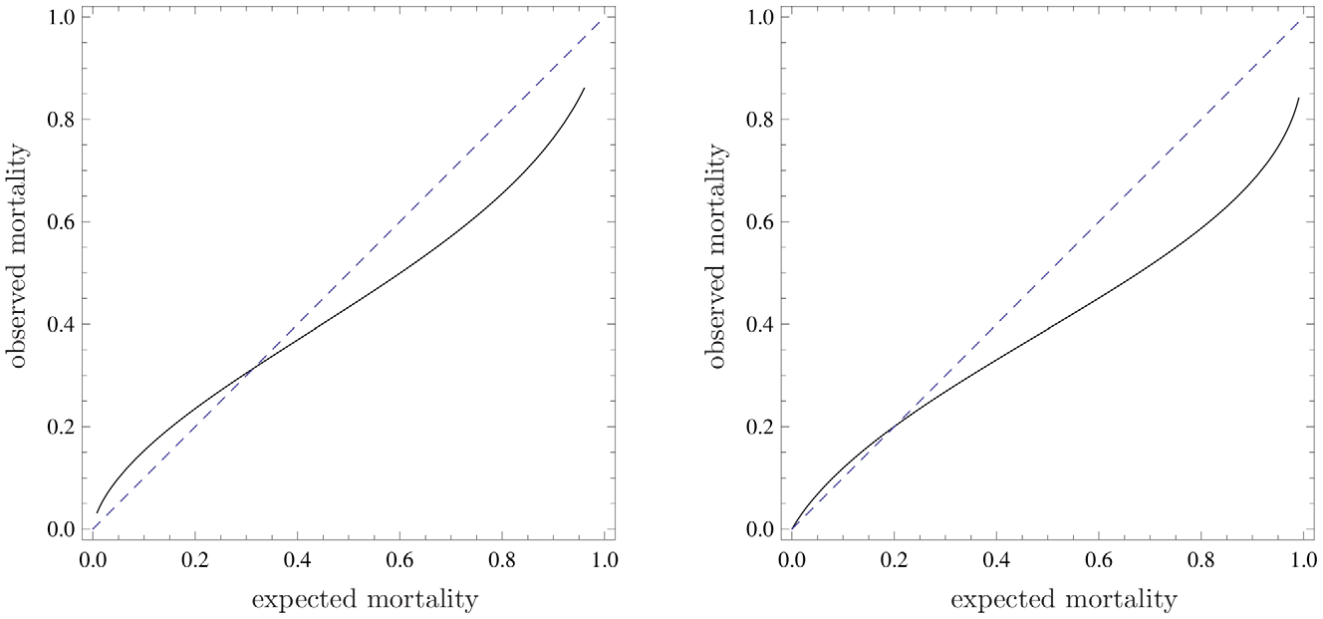
Calibration functions (*solid line*) compared to the bisector (*dashed line*) for the two discussed examples. The stopping criterion yielded 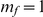 for the left curve and 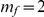 for the right one. To avoid extrapolation the curve have been plotted in the range of mortality where data are present. Refer to the caption of Fig. 1 for information about the data sets.

One approach to obtain more detailed information about the range of probabilities in which the model does not calibrate well, is to plot the calibration function of Eq. (5), built through the estimated coefficients 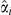, with 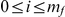, where 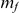 is fixed by the above described procedure. In Fig. 2, we plot such a curve for our examples in the range of expected probability for which observations are present, in order to avoid extrapolation. The model calibrates well when the calibration curve is close to the bisector. This curve is clearly more informative than the traditional calibration plot of expected against observed outcomes, averaged over subgroups (Fig. 1). In fact, spurious effects related to statistical noise due to low populated subgroups (in high risk deciles) are completely suppressed in this new plot. However, no statistically meaningful information concerning the deviation of the curve from the bisector has yet been provided.

### The calibration belt

To estimate the degree of uncertainty around the calibration curve, we have to compute the curve's confidence belt. In general, given a confidence level 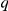, by performing lots of experiments, the whole unknown true curve 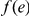 will be contained in the confidence belt in a fraction 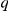 of experiments. The problem of drawing a confidence band for a general logistic response curve (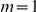) has been solved in [10], [11]. In Appendix S1, the analysis of [10] is generalized to the case in which 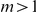. In this section we report only the result.

Determining a confidence region for the curve 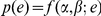 is equivalent to determining a confidence region in the 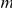-dimensional space of parameters 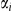. This is easy once one notes that, for large 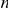, the estimated 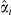, obtained by maximizing the likelihood of Eq. (6), have a multivariate normal distribution with mean values 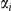, variances 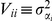, and covariances 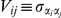 (see Eq. (S2) in Appendix S1).

Given a confidence level 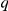, it is possible to show (see Appendix S1) that the confidence band for 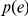 is
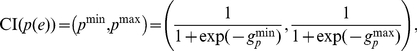
(9)where the confidence interval of the logit 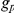 is
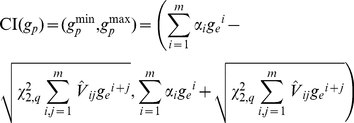
(10)and 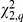 is the inverse of the 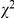 cumulative distribution with 2 degrees of freedom. The above the variances denotes that the values are estimated through the maximum likelihood method.

It is worth noting the one-to-one correspondence between this procedure to build the confidence band and the Wald test applied to the set of parameters 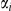. In fact, when the test 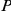-value is less than 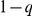, the band at 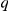 confidence level does not include the bisector and *vice versa*.

**Figure 3:**
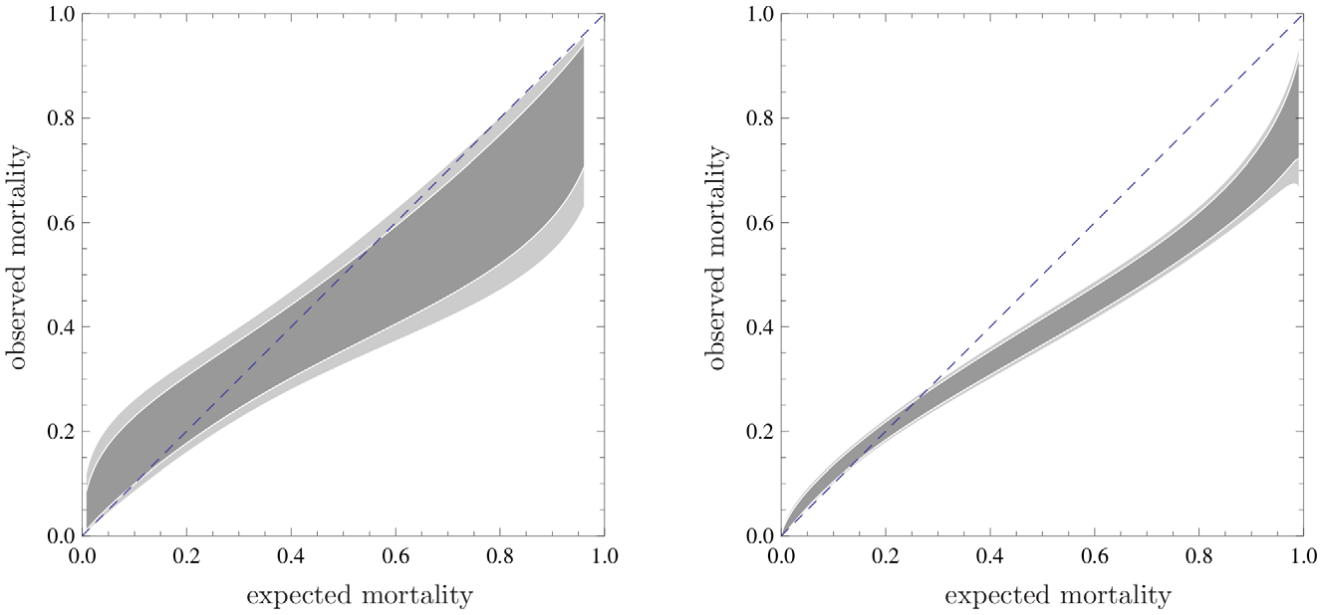
Calibration belts for the two discussed examples at two confidence levels. 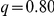
 (*dark shaded area*) and 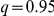 (*light shaded area*); 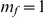 for the first example (left panel), 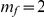 for the second (right panel). bisector (*dashed line*). As in Fig. 2, the calibrations bands have been plotted in the range of mortality where data are present. Refer to the caption of Fig. 1 for information about the data sets.

We are now able to plot the confidence belt to estimate the observed probability 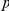, as a function of the estimated probability 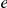, given by a reference model. Since the parameters of the calibration curve and belt are estimated through a fitting procedure, in order to prevent incorrect extrapolation, one must not extend them outside the range of expected probability 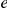 in which observations are present. In Fig. 3 we plot two confidence belts, for both examples, using 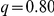 (inner belt, dark gray) and 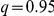 (outer belt, light gray). Statistically significant information on the region where the calibration curve calibrates poorly can now be derived from this plot, where the bisector is not contained in the belt.

In the first example (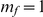), the confidence belts do not contain the bisector for expected mortality values higher than 0.56 (80% confidence level) and 0.83 (95% confidence level). This clarifies the result of the Hosmer–Lemeshow tests which have already highlighted the poor miscalibration of the model for the particular ICU. Now it is possible to claim with confidence that this miscalibration corresponds to better performance of the studied ICU compared to the national average for high severity patients.

In the second example, given the larger sample, the number of significant parameters is 3 (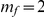) and the information provided by the calibration belt is very precise, as proven by the very narrow bands. From the calibration belt, the observed mortality is lower than the expected one when this is greater than 0.25, while the model is well calibrated for low-severity patients. The lower-than-expected mortality is not surprising and can be attributed to improvements of the quality of care since SAPS II was developed, about 15 years before data collection.

## Discussion

Calibration, which is the ability to correctly relate the real probability of an event to its estimation from an external model, is pivotal in assessing the validity of predictive models based on dichotomous variables. This problem can be approached in two ways. First, by using statistical methods which investigate the overall calibration of the model with respect to an observed sample. This is the case with the SMR, the Hosmer–Lemeshow statistics, and the Cox calibration test. As shown in this paper, all these statistics have drawbacks that limit their application as useful tools in quality of care assessment. The aim of the second approach is to localize possible miscalibration as a function of expected probability. An easy but misleading way to achieve this target is to plot averages of observed and expected probability over subsets. As illustrated above, this procedure might lead to non-informative or even erroneous conclusions.

We propose a solution to assess the dependence of calibration on the expected probability, by fitting the observed data with a very general calibration function, and plotting the corresponding curve. This method also enables confidence intervals to be computed for the curve, which can be plotted as a calibration belt. This approach allows to finely discriminate the ranges in which the model miscalibrates, in addition to indicating the direction of this phenomenon. This method thus offers a substantial improvement in the assessment of quality of care, compared to other available tools.

## Supporting Information

Appendix S1
**Computation of the confidence band.** In this Appendix, we compute the confidence band for the calibration curve. By generalizing the procedure given in [10] to the case in which 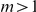, we demonstrate the results reported in Eqs. (9) and (10).(PDF)
