# Supplementary material for: Calibration Belt for Quality-of-Care Assessment Based on Dichotomous Outcomes
Source: PLoS One. 2011 Feb 23;6(2):e16110. doi: 10.1371/journal.pone.0016110 (PMC3043050; doi:10.1371/journal.pone.0016110)
Supplement: Appendix S1 — Computation of the confidence band. In this Appendix, we compute the confidence band for the calibration curve. By generalizing the procedure given in [10] to the case in which , we demonstrate the results reported in Eqs. (9) and (10). (PDF) [file pone.0016110.s001.pdf]

Appendix S1. Computation of the confidence band

The problem of drawing a confidence band for a general logistic response curve was solved in [1,2]. Here we generalize the procedure given in [1] to the case in which $m > 1$. In the limit of large n, the estimated $\hat{\alpha}_i$, obtained by maximizing the likelihood of Eq. (6), have a multivariate normal distribution with mean

$$\mathbf{A} = \begin{pmatrix} \alpha_0 \\ \alpha_1 \\ \cdots \\ \alpha_m \end{pmatrix} \tag{S1}$$

and covariance matrix V

$$\mathbf{V} = \begin{pmatrix} \sigma^2_{\alpha_0} & \sigma_{\alpha_0\alpha_1} & \cdots & \sigma_{\alpha_0\alpha_m} \\ \sigma_{\alpha_0\alpha_1} & \sigma^2_{\alpha_1} & \cdots & \sigma_{\alpha_1\alpha_m} \\ \cdots & \cdots & \cdots & \cdots \\ \sigma_{\alpha_0\alpha_m} & \sigma_{\alpha_1\alpha_m} & \cdots & \sigma^2_{\alpha_m} \end{pmatrix}. \tag{S2}$$

Since $\mathbf{V}$ is unknown, we must estimate it from the data. Its estimator $\hat{\mathbf{V}}$ is fixed by

$$(\hat{V}^{-1})_{ij} = - \left. \frac{\partial^2 l}{\partial\alpha_i \partial\alpha_j} \right|_{\alpha_i = \hat{\alpha}_i}. \tag{S3}$$

Replacing $\mathbf{V}$ with $\hat{\mathbf{V}} \approx \mathbf{V}$, it is possible to show that $(\hat{\mathbf{A}} - \mathbf{A})'\hat{\mathbf{V}}^{-1}(\hat{\mathbf{A}} - \mathbf{A})$ is approximately distributed as a χ^2 with 2 degrees of freedom, so that, given a confidence level q:

$$\mathcal{P}\left[(\hat{\mathbf{A}} - \mathbf{A})'\hat{\mathbf{V}}^{-1}(\hat{\mathbf{A}} - \mathbf{A}) \le \chi^2_{2,q}\right] = q. \tag{S4}$$

Using the Cauchy-Schwartz inequality on the vectors $\hat{\mathbf{A}} - \mathbf{A}$ and $\hat{\mathbf{V}}\mathbf{G}$, with the scalar product defined by $\langle \mathbf{X}, \mathbf{Y} \rangle \equiv \mathbf{X}'\hat{\mathbf{V}}\mathbf{Y}$ ($\hat{\mathbf{V}}$ is symmetric), one gets

$$(\hat{\mathbf{A}} - \mathbf{A})'\hat{\mathbf{V}}^{-1}(\hat{\mathbf{A}} - \mathbf{A}) \ge \frac{[(\hat{\mathbf{A}} - \mathbf{A})'\mathbf{G}]^2}{\mathbf{G}'\hat{\mathbf{V}}\mathbf{G}} \tag{S5}$$

for any vector $\mathbf{G}$, in particular for $\mathbf{G} = (1, g_e, \ldots, {g_e}^m)$. Using the last equation in Eq. (S4), the confidence interval for $g_p = \mathbf{A}'\mathbf{G} = \sum_{i=1}^m \alpha_i \, {g_e}^m$ at the confidence level q is

$$\begin{aligned} \mathrm{CI}(g_p) = (g_p^{\min}, g_p^{\max}) = \mathrm{CI}(\mathbf{A}'\mathbf{G}) &= \left(\hat{\mathbf{A}}'\mathbf{G} - \sqrt{\chi^2_{2,q}\mathbf{G}'\hat{\mathbf{V}}\mathbf{G}}, \hat{\mathbf{A}}'\mathbf{G} + \sqrt{\chi^2_{2,q}\mathbf{G}'\hat{\mathbf{V}}\mathbf{G}}\right) \\ &= \left(\sum_{i=1}^m \alpha_i \, {g_e}^i - \sqrt{\chi^2_{2,q} \sum_{i,j=1}^m \hat{V}_{ij} \, {g_e}^{i+j}}, \sum_{i=1}^m \alpha_i \, {g_e}^i + \sqrt{\chi^2_{2,q} \sum_{i,j=1}^m \hat{V}_{ij} \, {g_e}^{i+j}}\right). \end{aligned} \tag{S6}$$

The confidence band for $p(e)$ is obtained from Eq. (5), since the logistic function is monotonically increasing:

$$\mathrm{CI}(p(e)) = (p^{\min}, p^{\max}) = \left(\frac{1}{1 + \exp(-g_p^{\min})}, \frac{1}{1 + \exp(-g_p^{\max})}\right). \tag{S7}$$

For the sake of completeness, we just mention that another different approach is possible in the computation of confidence bands [3], by simply connecting the single confidence limits of the mean values of $f(e)$, for each value of e. In this case, given a confidence level q, we can state that, having performed a large number of experiments, the true unknown value of the mean of $f(e)$, for every value of e, will lie within the confidence interval for the considered value of e, in a fraction q of the experiments. Note the difference with the presented method in which the whole curve (*i.e.* all its points simultaneously) is required to lie inside the confidence band in a fraction q of experiments.

In this alternative approach the confidence interval for $p = f(e)$ is determined for each value of e. Since $\hat{\alpha}_i$ have a multivariate normal distribution, $\hat{g}_p$ has a normal distribution with mean $\mathbf{A}'\mathbf{G}$ and variance

$$\sigma^2_{g_p} = \mathbf{G}'\mathbf{V}\mathbf{G} = \sum_{i,j=1}^m V_{ij} \, {g_e}^{i+j}. \tag{S8}$$

Therefore the variable $(\hat{g}_p - g_p)/\hat{\sigma}_{g_p}$, where $\hat{\sigma}_{g_p}$ is obtained by the above formula by replacing the variance $\mathbf{V}$ with its estimator $\hat{\mathbf{V}}$, is distributed as a Student t with $n-2$ degrees of freedom. The confidence interval for g_p is

$$\mathrm{CI}^t(g_p) \;=\; (g_p^{\min}, g_p^{\max}) \;=\; \mathrm{CI}^t(\mathbf{A}'\mathbf{G}) \;=\; \left(\hat{\mathbf{A}}'\mathbf{G} - t_{n-2,q}\sqrt{\mathbf{G}'\hat{\mathbf{V}}\mathbf{G}}, \hat{\mathbf{A}}'\mathbf{G} + t_{n-2,q}\sqrt{\mathbf{G}'\hat{\mathbf{V}}\mathbf{G}}\right). \quad \text{(S9)}$$

We use the superscript t to denote the Student distribution. However, provided n is large enough (say, greater than 50), as it should be in the situations in which this method would be applied, the Student distribution can be safely approximated by the Normal distribution ($t_{n-2,q} \approx z_q$). The confidence band for $p(e)$ is obtained as above from Eqs. (5) and (S9).

References

[1] Hauck W (1983) A note on confidence bands for the logistic response curve. The American Statistician 37: 158-160.

[2] Brand R, Pinnock D, Jackson K (1973) Large sample confidence bands for the logistic response curve and its inverse. The American Statistician 27: 157-160.

[3] Montgomery D, Peck E, Vining G (2001) Introduction to linear regression analysis. New York, NY: Wiley, 3rd edition.
